# Morphometric and structural analysis of Florida manatee spermatozoa

**DOI:** 10.1002/ar.24645

**Published:** 2021-05-06

**Authors:** Jonathan R. Cowart, Danielle M. Collins, Daniel L. Stanton, Gerhard van der Horst, Iskande V. Larkin

**Affiliations:** ^1^ Aquatic Animal Health Program College of Veterinary Medicine, University of Florida Gainesville Florida USA; ^2^ Department of Animal Sciences College of Agricultural and Life Sciences, University of Florida Gainesville Florida USA; ^3^ University of Florida Institute of Food and Agricultural Sciences, Citrus Research and Education Center Lake Alfred Florida USA; ^4^ Department of Medical Bioscience University of the Western Cape Bellville South Africa

**Keywords:** CASA, Florida manatee, reproduction, SEM, sperm, sperm morphology, sperm ultrastructure, TEM

## Abstract

Sperm characteristics, such as sperm morphology and sperm morphometry are important in assessing sperm quality. This is especially important for the management and conservation of endangered and exotic species, like the Florida manatee, where information of this nature is extremely limited. In this study, we fill this knowledge gap to better understand the reproductive physiology of Florida manatees by conducting the first extensive analysis of sperm morphometry and ultrastructure. Sperm were retrieved from the vas deferens of nine recently deceased Florida manatees. Computer‐aided sperm morphology analysis (CASMA) was used for morphometric analysis and laser‐scanning confocal microscopy and electron microscopy were used for structural and ultrastructural characterization. Our findings reveal new morphometric and structural data for the Florida manatee spermatozoon. Twelve morphometric features of Florida manatee sperm were quantified with some approximately 1.5–2 times larger than those previously reported. Ultrastructurally, the Florida manatee spermatozoon followed a mammalian structural pattern with an ovate‐shaped head, midpiece containing 84–90 mitochondria, and a flagellum. However, unique ultrastructural features were identified. Distinct, rectangular‐like enlargement of four outer dense fibers surrounding the axoneme was evident, which may provide additional tensile strength to counteract the forces on sperm transiting the female reproductive tract. Likewise, strong localization of F‐actin fibers within the midpiece may function to maintain sperm integrity within the female reproductive tract. These findings highlight the potential effects of sexual selective pressures on sperm size and structure in the Florida manatee and provide avenues for research on the occurrence of sperm competition in this species.

## 1 INTRODUCTION

The Florida manatee (*Trichechus manatus latirostris*), a subspecies of the West Indian manatee, is one of the four sirenian species currently listed as “vulnerable” on the IUCN red list. Although population numbers have increased over the past decade (Kleen & Breland, 2014; Martin et al., 2015; Semeyn et al., 2011), this species faces high annual injury and mortality due to intense natural and anthropogenic threats. These threats underlie a critical need for a better understanding of key life history parameters. This includes a particular focus on reproduction as it has important implications for the continued survival and growth of the population. Despite being one of the most studied sirenian species, information about the reproductive physiology of the Florida manatee, especially that of male manatees, is still limited. Therefore, further assessment of different reproductive parameters is necessary to gain a better understanding of reproduction in this species.

In order to contribute to an understanding of male fertility in the Florida manatee it is important to investigate sperm characteristics, including sperm morphology, morphometry, and structure. While there is great diversity in sperm morphology and morphometry amongst different species, the structural composition of the spermatozoon is relatively conserved across mammalian taxa. Each spermatozoon is composed of a head with a nucleus and a flagellum, divided into a midpiece containing mitochondria, a principal piece, and an endpiece. Each component of the spermatozoon has a specific function related to fertilization and the morphological and structural organization of the spermatozoon provides essential information about the functional roles of each component and their potential relationship with reproductive success. Likewise, these characteristics also yield valuable insight into the reproductive physiology of a species, the reproductive health and fitness of individual males, and the evolution of reproductive systems and phylogenetic relationships within and among species (Beilis, Cetica, & Merani, 2000).

This information is especially important for endangered and exotic species, such as the Florida manatee, where information regarding the spermatozoon is almost non‐existent. To date, only one study by Miller, Dougherty, Decker, and Bossart (2001) has primarily focused on the Florida manatee spermatozoon. Unfortunately, the resulting morphometric and ultrastructural data are limited thus restricting insights into the relationship between sperm form and function in this species and highlighting the critical need to build upon this information to better understand fertility in the Florida manatee.

Accordingly, we investigated the morphology, morphometry, and ultrastructure of the Florida manatee spermatozoon by means of light microscopy, computer‐aided sperm morphology analysis (CASMA), scanning and transmission electron microscopy (SEM and TEM), and laser‐scanning confocal microscopy (LSCM). The results of this study are important in providing baseline information as well as contributing to parameters important in sperm quality assessment for Florida manatees.

## 2 MATERIALS AND METHODS

### 2.1 Sperm collection

Sperm were collected from the vas deferens of nine recently deceased (<24–48 hr postmortem), sexually mature, male Florida manatees retrieved by the Florida Fish and Wildlife Conservation Commission Marine Mammal Pathobiology Laboratory in St. Petersburg, Florida. Causes of death for males in this study included brevetoxicosis (red tide), trauma from watercraft collisions, other human interactions, and undetermined causes (Table 1). Because of the acute nature of death for each male, it was believed that the cause of death had no likely effect on sperm quality. Sperm retrieval was achieved by making a transverse incision through the vas deferens and compressing it to “milk” sperm out where it was pipetted into 1 ml phosphate buffered saline (PBS). Sperm were then washed twice in PBS by centrifugation at 1653*g* for 8 min. For morphometric analysis, the resulting pellet was resuspended in PBS and a 10 μl aliquot of sperm was smeared on a glass slide and air‐dried overnight for subsequent staining. For ultrastructural analyses, the resulting pellet was fixed in either 2.5% glutaraldehyde in 0.1 M sodium cacodylate buffer or 4% paraformaldehyde and stored at 4°C until analysis. All samples were collected and analyzed under Federal Fish and Wildlife Permit #MA067116‐2 and in accordance with University of Florida Institutional Animal Care and Use Committee protocol #20180884.

**TABLE 1:** Individual male Florida manatee summary information

Animal ID	Total body length (cm)	Age classification	Cause of death
MNW18044	322	Adult	Undetermined
MSE17035	299	Adult	Human; other
MSW17121	270	Adult	Watercraft collision
MSW18221	298	Adult	Red tide
MSW18245	308	Adult	Red tide
MSW18248	248	Sub adult	Red tide
MNW18103	302	Adult	Red tide
MSW18249	328	Adult	Red tide
SWFTm1831b	292	Adult	Watercraft collision

*Note*: Summary information was obtained from the necropsy reports completed by the Florida Fish and Wildlife Conservation Commission Marine Mammal Pathobiology Laboratory for each individual manatee.

### 2.2 Morphometric and morphological analysis

Sperm morphometry was assessed using a modified SpermBlue^®^ staining protocol as described by van der Horst and Maree (2010). In short, slides were immersed in SpermBlue^®^ (Microptic, S.L., Barcelona, Spain) for 50 sec then gently rinsed in distilled water for 4 s to remove excess stain. Slides were air‐dried overnight and permanently mounted using dibutyl phthalate xylene (DPX, Sigma–Aldrich). A total of 200 sperm per sample were randomly analyzed for each of the morphometric parameters listed in Table 2. Only fully intact sperm were analyzed and all morphometric parameters were analyzed at ×400 total magnification by computer‐aided sperm morphology analysis (CASMA) using a Microptic Sperm Class Analyzer^®^ (SCA^®^) computer‐aided sperm analysis (CASA) system version 6.3 with the SCA^®^ morphology module. Sperm analyzed by CASMA were visually assessed by a single investigator (JC) to ensure correct thresholding and that no over‐ or underestimation of morphometric parameters occurred through the automated analysis. Improperly measured sperm were removed from analysis. Sperm morphology was also manually assessed using the same SpermBlue^®^ staining protocol described above for determining different morphological abnormalities including head abnormalities, proximal and distal cytoplasmic droplets, bent or coiled midpieces and tails, and detached heads (200 sperm per sample, ×600 total magnification).

**TABLE 2:** Morphometric parameters of Florida manatee sperm analyzed through computer‐aided sperm morphology analysis (CASMA)

Morphometric parameters	Formula
Head length (μm)	L
Head width (μm)	W
Head area (μm^2^)	A
Head perimeter (μm)	P
Midpiece length (μm)	–
Principal + end piece length (μm)	–
Flagellum length (μm)	–
Total length (μm)	–
**Shape index parameters:**	
Head ellipticity	L/W
Head elongation	(L – W)/(L + W)
Head roughness	4π (A/P^2^)
Head regularity	π (L * W/4A)

### 2.3 Ultrastructural analysis

Transmission electron microscopy (TEM) and scanning electron microscopy (SEM) were used to study the normal ultrastructure of Florida manatee sperm as well as morphological abnormalities. For SEM, samples fixed in 2.5% glutaraldehyde in 0.1 M sodium cacodylate buffer were filtered through 0.2 μm GTTP Isopore Membrane Filters (Merck Millipore Ltd., Tullagreen, Cork, Ireland), which were previously treated with a 1:10 poly‐L‐lysine solution (Sigma–Aldrich, St. Louis, MO) to assist in adherence to the filter. Filtered samples were then post‐fixed in Trump's fixative (Electron Microscopy Sciences, Hatfield, PA). Fixed cells were processed with the aid of a Pelco BioWave laboratory microwave (Ted Pella, Redding, CA). Samples were washed in 0.1 M sodium cacodylate, pH 7.22, post fixed with sodium cacodylate buffered 2% osmium tetroxide (OsO_4_), water washed, and dehydrated in a graded ethanol series (25% through 100%, with 25% increments). Samples were critical point dried using the Tousimis Autosamdri‐815 critical point dryer (Tousimis, Rockville, MD). Once dried, samples were mounted onto 12 mm carbon conductive adhesive tabs and aluminum stubs (Electron Microscopy Sciences, Hatfield, PA). Mounted samples were then sputter coated with gold/palladium using the Denton Desk V sputter coater (Denton Vacuum, Moorestown, NJ). Samples were imaged with a Hitachi SU‐5000 FE‐SEM (Hitachi High Technologies in America, Greenville, SC) with use of EM Wizard software. Additional images and preliminary measurements to validate the morphometric results obtained through CASMA were also completed on a small subset of sperm analyzed by SEM. Images and measurements were acquired using a Hitachi S‐4000 SEM (Hitachi High Technologies in America, Greenville, SC) and PCI imaging software (Quartz Imaging Corp., Vancouver, BC).

For TEM, fixed samples in 2.5% glutaraldehyde in 0.1 M sodium cacodylate buffer were centrifuged at 2650*g* for 10 min and the fixative removed. The resulting sperm pellet was encapsulated in 3% agarose and washed in 0.1 M sodium cacodylate, pH 7.22, post fixed with buffered 2% OsO_4_, water washed, and dehydrated in a graded ethanol series (25%–100%, with 5% increments). Dehydrated samples were infiltrated in LRWhite Medium and Z6040 embedding primer (Electron Microscopy Sciences, Hatfield, PA) in 50% and 100%, respectively, and cured at 60°C for 48 hr. Ultra‐thin sections were collected on carbon coated Formvar 100 mesh copper grids (Electron Microscopy Sciences, Hatfield, PA) and post‐stained with 2% aqueous uranyl acetate for 6 min and then Reynold's lead citrate for 4 min. Sections were examined with a FEI Tecnai G2 Spirit Twin TEM (FEI Corp., Hillsboro, OR) and digital images were acquired with a Gatan UltraScan 2 k × 2 k camera and Digital Micrograph software (Gatan Inc., Pleasanton, CA).

### 2.4 Laser‐scanning confocal microscopy

Laser‐scanning confocal microscopy (LSCM) was used to assess cytoskeletal and nuclear aspects of Florida manatee sperm. Samples fixed in 4% paraformaldehyde were pelleted by centrifugation at 1900*g* for 8 min at room temperature and the fixative removed. Sperm were then washed twice in PBS by centrifugation at 1900*g* for 8 min to remove remaining fixative. Samples were treated with rabbit anti‐alpha tubulin (1:250; ab52866, Abcam) in PBS with 1% bovine serum albumin (BSA) and 0.1% Triton X‐100 overnight at 4°C. Samples were washed twice in PBS and incubated in Alexa Fluor 568 goat anti‐rabbit secondary antibody (1:500; A11011, Invitrogen), phalloidin‐iFluor 488 (1:250; ab176753, Abcam), and DAPI (1:500; D1306, Invitrogen) in PBS/1% BSA/ 0.1% Triton X‐100 overnight at 4°C. Specificity controls included anti‐alpha tubulin primary antibody only and phalloidin‐iFluor 488 antibody only. Omission of the anti‐alpha tubulin primary antibody was used as a control for non‐specific staining of the secondary antibody. Omission of primary antibodies was used as a negative control. Samples were pelleted and washed twice in PBS and mounted on a slide with vectashield (Vector laboratories, Burlingame, CA). Sperm were imaged using a Leica SP8 LSCM (Leica Microsystems, Wetzlar, Germany) equipped with a 405‐diode laser, 488‐argon laser, and a 561‐HeNe laser. Images were captured using LASX software (Leica Microsystems, Wetzlar, Germany).

### 2.5 Statistical analysis

Morphometric data were characterized by descriptive statistics including means, standard deviations, and ranges for each parameter. All analyses were calculated using R software version 3.6.1.

## 3 RESULTS

### 3.1 Sperm morphometry

**FIGURE 1:**
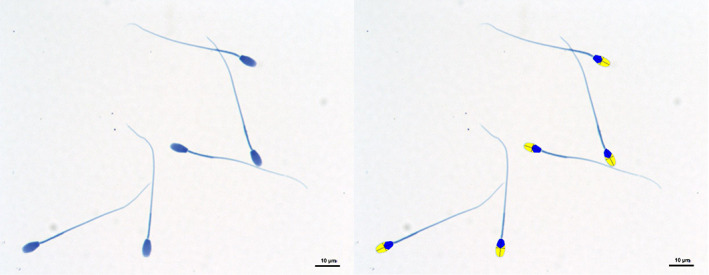
Computer‐aided sperm morphometric analysis of Florida manatee sperm stained with SpermBlue^®^. (Left) Sperm stained with SpermBlue^®^ before morphometric analysis. (Right) Sperm analyzed by the SCA^®^ computer‐aided sperm analysis system showing accurate thresholding of the acrosome (yellow) and head (blue)

A total of 1,800 sperm from nine male Florida manatees were analyzed through computer‐aided sperm morphology analysis (CASMA). Overall mean morphometric measurements of Florida manatee sperm were as follows: head length (x̅ = 7.52 ± 0.34 μm), head width (x̅ = 3.44 ± 0.20 μm), head area (x̅ = 24.60 ± 3.15 μm^2^), head perimeter (x̅ = 15.93 ± 0.88 μm), head ellipticity (x̅ = 2.19 ± 0.13), head elongation (x̅ = 0.37 ± 0.03), head roughness (x̅ = 1.22 ± 0.16), head regularity (x̅ = 0.83 ± 0.08), midpiece length (x̅ = 10.4 ± 0.37 μm), principal piece + end piece length (x̅ = 41.08 ± 1.37 μm), flagellum length (x̅ = 51.49 ± 1.42 μm), and total sperm length (x̅ = 58.98 ± 1.53 μm). Mean morphometric measurements for each parameter for each individual sample are reported in Table 3. Illustration of accurate thresholding of the acrosome and head conducted by CASMA is depicted in Figure 1.

**TABLE 3:** Mean values of measured morphometric parameters for each individual Florida manatee sample

Animal ID	Morphometric parameters											
	Head length (μm)	Head width (μm)	Head area (μm^2^)	Head perimeter (μm)	Head Ellipticity	Head elongation	Head roughness	Head regularity	Midpiece length (μm)	Principal + end piece (μm)	Flagellum length (μm)	Total length (μm)
MNW18044	7.38 ± 0.36	3.36 ± 0.22	23.22 ± 2.82	15.69 ± 0.93	2.20 ± 0.15	0.37 ± 0.03	1.19 ± 0.14	0.85 ± 0.07	10.26 ± 0.47	39.91 ± 1.31	50.17 ± 1.36	57.48 ± 1.43
MSE17035	7.41 ± 0.29	3.59 ± 0.14	25.17 ± 3.03	15.87 ± 0.76	2.07 ± 0.09	0.35 ± 0.02	1.26 ± 0.16	0.84 ± 0.08	10.36 ± 0.36	41.29 ± 1.21	51.65 ± 1.25	59.00 ± 1.36
MSW17121	7.73 ± 0.25	3.43 ± 0.14	25.95 ± 2.19	16.28 ± 0.72	2.26 ± 0.09	0.39 ± 0.02	1.24 ± 0.14	0.81 ± 0.06	10.62 ± 0.30	41.43 ± 0.89	52.04 ± 0.91	59.82 ± 0.96
MSW18221	7.43 ± 0.26	3.48 ± 0.17	24.96 ± 3.09	15.86 ± 0.77	2.14 ± 0.10	0.36 ± 0.02	1.25 ± 0.17	0.82 ± 0.09	10.53 ± 0.30	40.88 ± 1.12	51.41 ± 1.16	58.81 ± 1.21
MSW18245	7.62 ± 0.31	3.56 ± 0.21	25.95 ± 3.33	16.12 ± 0.85	2.15 ± 0.11	0.36 ± 0.02	1.26 ± 0.17	0.83 ± 0.08	10.40 ± 0.34	41.81 ± 0.95	52.21 ± 0.93	59.81 ± 1.01
MSW18248	7.57 ± 0.32	3.27 ± 0.21	24.25 ± 2.71	15.82 ± 0.96	2.32 ± 0.13	0.40 ± 0.02	1.22 ± 0.16	0.81 ± 0.07	10.47 ± 0.31	41.74 ± 0.97	52.20 ± 0.91	59. 70 ± 1.02
MNW18103	7.56 ± 0.39	3.42 ± 0.19	23.63 ± 3.65	16.00 ± 0.93	2.21 ± 0.11	0.38 ± 0.02	1.16 ± 0.15	0.87 ± 0.09	10.40 ± 0.35	41.74 ± 1.70	52.14 ± 1.71	59.76 ± 1.73
MSW18249	7.39 ± 0.30	3.36 ± 0.15	23.65 ± 2.96	15.69 ± 0.85	2.20 ± 0.10	0.38 ± 0.02	1.21 ± 0.16	0.83 ± 0.09	10.41 ± 0.29	40.52 ± 0.95	50.94 ± 1.00	58.28 ± 1.02
SWFTm1831b	7.63 ± 0.34	3.49 ± 0.19	24.63 ± 3.17	16.09 ± 0.92	2.19 ± 0.12	0.37 ± 0.02	1.20 ± 0.16	0.86 ± 0.08	10.20 ± 0.39	40.45 ± 1.53	50.65 ± 1.58	58.20 ± 1.67
Mean	7.52 ± 0.34	3.44 ± 0.20	24.60 ± 3.15	15.93 ± 0.88	2.19 ± 0.13	0.37 ± 0.03	1.22 ± 0.16	0.83 ± 0.08	10.41 ± 0.37	41.08 ± 1.37	51.49 ± 1.42	58.98 ± 1.53
Range	5.73–9.36	2.75–4.33	16.34–36.36	13–19.03	1.80–2.77	0.29–0.47	0.89–1.95	0.57–0.97	8.04–11.65	34.68–60	44.95–70.32	50.78–77.81

*Note*: Values are reported as mean ± *SD*.

Scanning electron microscopic measurements on a limited subset of sperm (*n* = 20) from three different males yielded similar morphometric measurements as was acquired with CASMA: head length (x̅ = 7.21 ± 0.45 μm), head width (x̅ = 3.38 ± 0.29 μm), midpiece length (x̅ = 10.53 ± 0.45 μm), principal + end piece length (x̅ = 41.44 ± 1.39 μm), tail length (x̅ = 51.84 ± 1.69 μm), and total length (x̅ = 59.25 ± 1.82 μm). The enhanced magnification and resolution provided by SEM allowed for additional morphometric parameters to be measured including connecting piece length (x̅ = 0.46 ± 0.15 μm), midpiece width (x̅ = 0.72 ± 0.17 μm), principal piece length (x̅ = 39.03 ± 1.50 μm), principal piece width (x̅ = 0.61 ± 0.11 μm), end piece length (x̅ = 2.38 ± 0.24 μm), and end piece width (x̅ = 0.20 ± 0.01 μm).

### 3.2 Sperm ultrastructure

**FIGURE 2:**
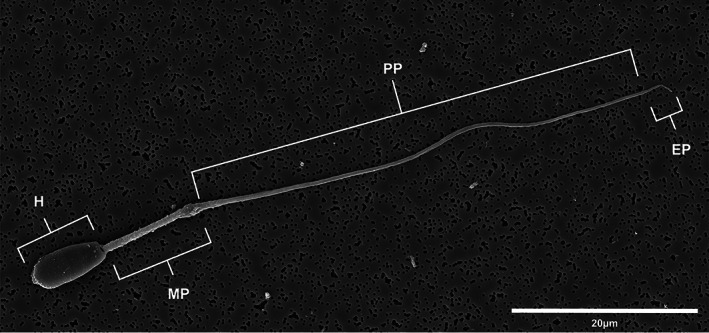
Scanning electron micrograph of a Florida manatee spermatozoon. All structural components typically seen in mammalian sperm are shown including the head (H) and flagellum, which is composed of the midpiece (MP), principal piece (PP), and the end piece (EP)

**FIGURE 3:**
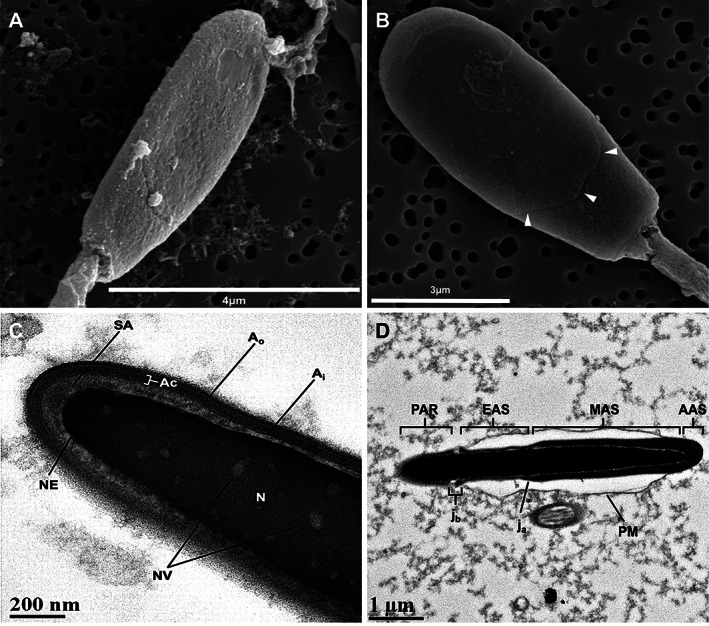
Scanning electron micrographs (a,b) and transmission electron micrographs (c,d) of the Florida manatee spermatozoon head. (a) Dorsoventrally flattened and ovate shaped spermatozoon head. (b) Equatorial region (white arrows) of spermatozoon head observed at the bottom of the acrosomal cap. (c) Longitudinal section of the spermatozoon head showing the nucleus (N), nuclear vacuoles (NV), nuclear envelope (NE), acrosome (Ac) composed of the inner acrosomal membrane (A_i_) and outer acrosomal membrane (A_o_), and subacrosomal space (SA). (d) Longitudinal section of the spermatozoon head showing the apical acrosomal segment (AAS), main acrosomal segment (MAS), equatorial acrosomal segment (EAS), and post‐acrosomal region (PAR). The junction between the MAS and EAS (j_a_) and between the EAS and PAR (j_b_) are also shown as is the plasma membrane (PM). Separation of plasma membrane from the sperm head is likely an artifact of sample collection and/or processing

The Florida manatee spermatozoon is composed of a head and a flagellum made up of a midpiece, principal piece, and end piece (Figure 2). The spermatozoon head was flattened dorsoventrally and ovate in shape (Figure 3a) with an acrosome that covered approximately 75% of the anterior portion of the spermatozoon head (Figure 3b). The nucleus was composed of dense chromatin, which was nearly homogenous and contained small nuclear vacuoles distributed evenly throughout the head (Figure 3c). The spermatozoon head was anteriorly covered by a pronounced acrosome, which formed a cap‐like structure over the nucleus. The acrosome typically contained an inner and outer acrosomal membrane with a subacrosomal space (Figure 3c). The entire head, including the nucleus and acrosome, was covered by a plasma membrane (Figure 3d). Three distinguishable segments of the acrosome were identified including the apical acrosomal segment, the main acrosomal segment, and the equatorial acrosomal segment (Figure 3d). The apical and main acrosomal segments (the segments involved in the acrosome reaction) were electron dense and pronounced, covering approximately 50% of the apical portion of the spermatozoon head. The acrosome abruptly thinned at the equatorial region representing the equatorial acrosomal segment. A distinct boundary between the acrosome and post‐acrosomal region was observed (Figure 3d).

**FIGURE 4:**
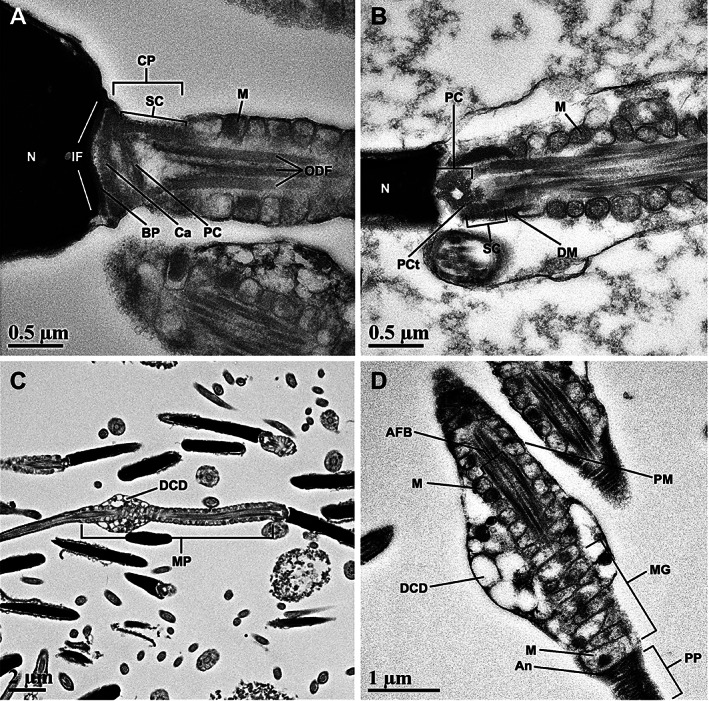
Transmission electron micrographs of the Florida manatee spermatozoon connecting piece (a,b) and midpiece (c,d). (a) Longitudinal section of spermatozoon showing the posterior portion of the nucleus (N), the connecting piece (CP), and the anterior portion of the midpiece. Structural components of the connecting piece include: implantation fossa (IF), basal plate (BP), capitulum (Ca), proximal centriole (PC), and segmented columns (SC). Mitochondria (M) and outer dense fibers (ODF) are observed within the midpiece. (b) Longitudinal section of spermatozoon showing the nucleus (N), proximal centriole (PC), tubular triplets of the proximal centriole (PCt), electron dense material (DM), mitochondria (M). (c) Longitudinal section of the midpiece (MP), which exhibits a distal cytoplasmic droplet (DCD). (d) Longitudinal section through midpiece showing the axial fiber bundle (AFB), plasma membrane (PM), mitochondria (M), distal cytoplasmic droplet (DCD), mitochondrial gyres (MG), mitochondria (M), annulus (An), and anterior portion of the principal piece (PP)

The connecting piece, which represents the transition between the head and the midpiece, was similar to that of other mammalian sperm. At the caudal nuclear surface, the implantation fossa, where the internal filaments of the tail anchor into the head, was distinguished by its characteristic shallow concave depression (Figure 4a). The basal plate was located posterior and adherent to the nuclear envelope and appeared to be confined to the area of the implantation fossa with no observed extensions beyond these limits (Figure 4a). The capitulum was observed at the anterior region of the connecting piece, posterior to the basal plate. Segmented columns extended from the capitulum and connected to the outer dense fibers (ODFs; Figure 4a). Electron dense material was observed in some sections along the periphery of the segmented columns, between the segmented columns and the plasma membrane, and extending from the proximal end of the mitochondria within the midpiece (Figure 4b). A proximal centriole was observed in the apical portion of the connecting piece between the segmented columns, posterior to the basal plate and capitulum (Figure 4b). In some sections, tubular triplets of the proximal centriole could be identified.

**FIGURE 5:**
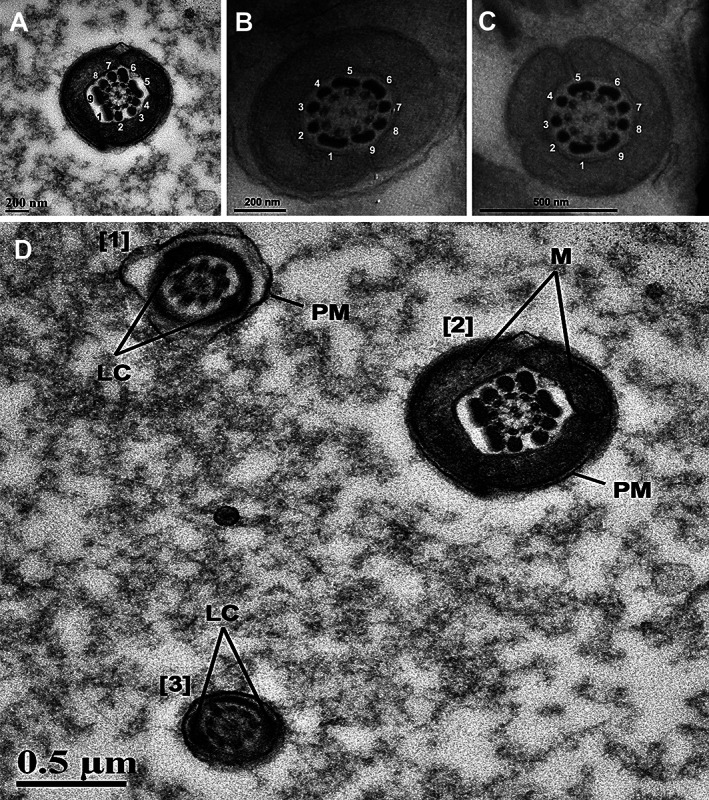
Transmission electron microscopic cross‐sectional profiles of the Florida manatee spermatozoon flagellum. (a–c) The 9 + 9 + 2 arrangement is evident with outer dense fibers 1, 5, 6, and 9 distinctly enlarged and morphologically rectangular‐like. Three cross sectional profiles from different males are provided as validity of the enlargement of the four outer dense fibers in this species. (d) Different regions of the Florida manatee spermatozoon flagellum. (1) cross section through the principal piece showing the longitudinal columns (LC) and plasma membrane (PM). (2) cross section through the midpiece as denoted by the mitochondria (M) surrounding the nine outer dense fibers and the axoneme. (3) cross section through the region transitioning from the principal piece to the end piece evidenced by the less than prominent longitudinal columns

The flagellum was composed of the midpiece, principal piece, and end piece. The axial fiber bundle of the midpiece was surrounded by approximately 84–90 mitochondria arranged end‐to‐end (Figure 4c). The mitochondria were oriented into 42–45 mitochondrial gyres wrapped helically around the ODFs of the midpiece and surrounded by a thin external plasma membrane (Figure 4d). Proximal and distal cytoplasmic droplets were observed along the midpiece in some sections (Figure 4c). Within the midpiece, a central pair of microtubules surrounded by nine uniformly spaced microtubule doublets, all surrounded by nine ODFs, were observed with ODFs 1, 5, 6, and 9 distinctly larger and elongated (unique rectangular‐like shape) in comparison to the other ODFs (Figure 5a–c). The annulus was present at the immediate caudal end of the mitochondrial sheath and was triangular in section with the rostral surface in direct contact with the last mitochondrial gyre and the caudal surface in close relation to the start of the fibrous sheath of the principal piece (Figure 4d).

The principal piece constituted the longest portion of the flagellum and was encapsulated by the fibrous sheath. The fibrous sheath was characterized by circumferential fibers extending around the tail between the dorsal and ventral longitudinal columns, which were positioned opposite each other and corresponded to ODFs 3 and 8 (Figure 5d). The end piece was observed at the posterior end of the principal piece and was short and thin compared to both the midpiece and the principal piece (Figure 2).

### 3.3 Localization of cytoskeletal proteins

**FIGURE 6:**
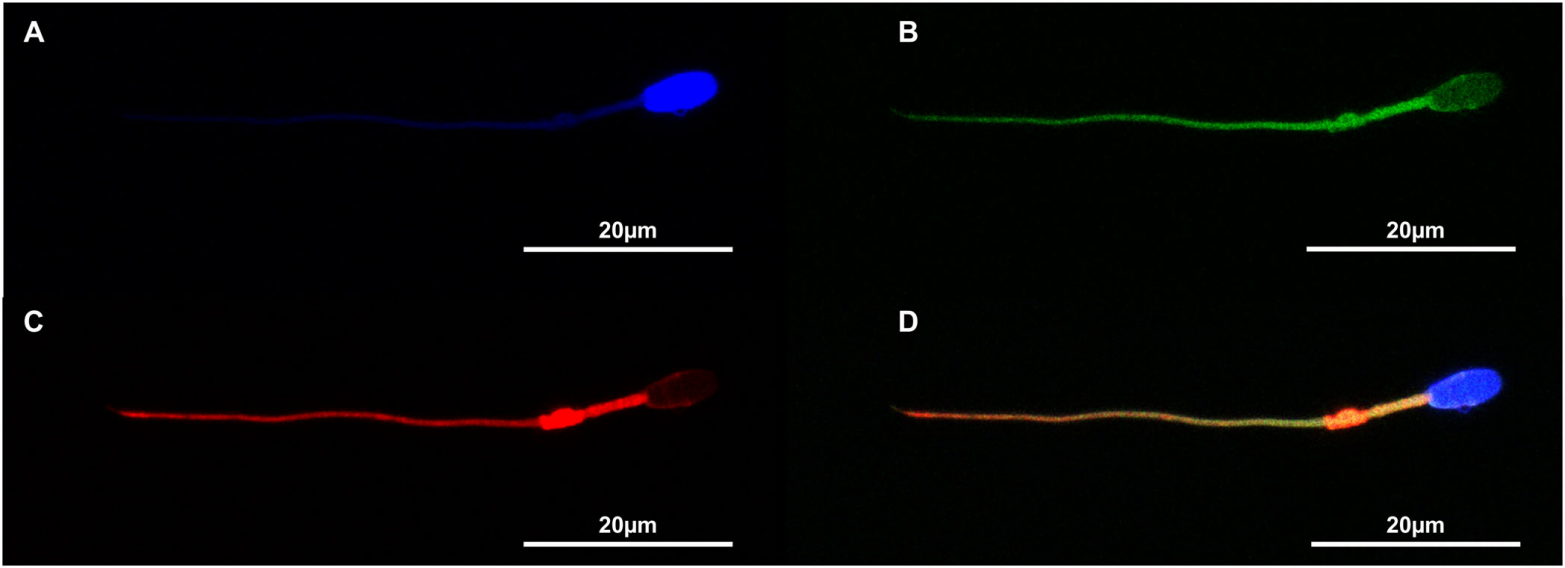
Immunofluorescent staining of Florida manatee spermatozoon with phalloidin‐iFluor 488 (F‐actin), anti‐alpha tubulin, and DAPI. (a) DAPI only. (b) F‐actin only. (c) Alpha‐tubulin only. (d) Overlaid frames of all three (×400 total magnification)

F‐actin filaments and microtubules were present throughout the spermatozoon including the spermatozoon head, midpiece, and flagellum; however, immunofluorescent staining intensity was variable for both (Figure 6). F‐actin displayed a moderate, spotty localization throughout the spermatozoon head with the highest localization at the interface of the spermatozoon head and connecting piece. The highest intensity localization was within the midpiece with decreasing localization throughout the flagellum. Staining localization of alpha‐tubulin within the spermatozoon head was similar to that of F‐actin, but more homogenous in appearance. The highest intensity of alpha‐tubulin staining was observed within the midpiece with slightly less throughout the remainder of the flagellum. For both F‐actin and alpha‐tubulin, there were two rings faintly localized along the anterior and posterior aspects of the equatorial region. Controls using the alpha‐tubulin primary antibody only, phalloidin‐iFluor 488 only, and secondary only controls did not produce any background or non‐specific staining effects and only labeled their respective targeted epitopes. Negative controls (no primary antibody) also yielded no staining effects either.

### 3.4 Morphological abnormalities

**FIGURE 7:**
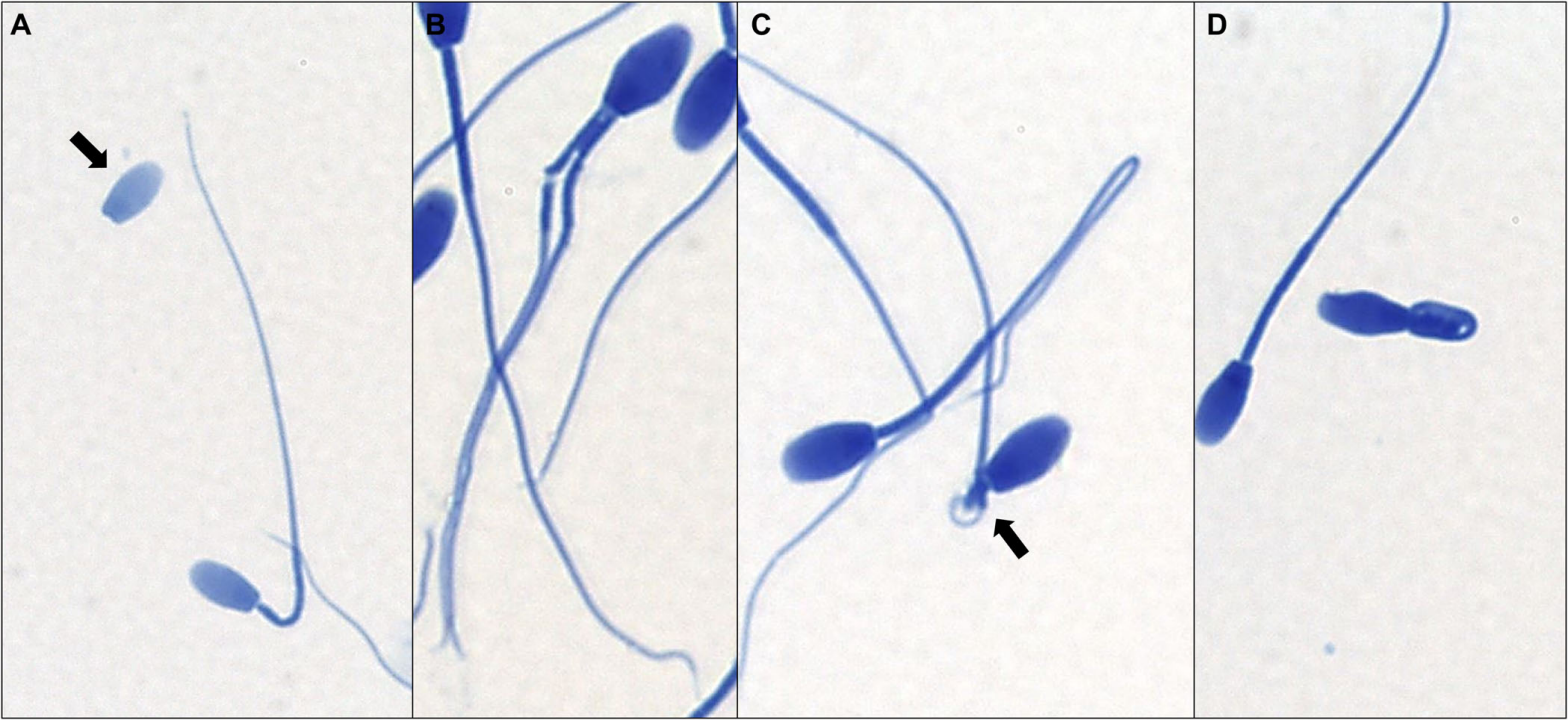
Sperm abnormalities observed during morphological analysis with SpermBlue^®^ staining. (a) Two spermatozoa with abnormalities: one with a detached head (arrow) and the other exhibiting a bent midpiece. (b) Spermatozoon with two tails with one tail broken at the midpiece. (c) Two spermatozoa with abnormalities: one exhibiting a hairpin loop at the distal end of the tail and the other exhibiting a bent midpiece (arrow). (d) Spermatozoon exhibiting Dag defect

Morphological abnormalities were observed across all samples analyzed with SpermBlue^®^ and electron microscopy. Mean values for sperm morphology analyzed with SpermBlue for each individual sample and across all samples are summarized in Table 4. Across all samples, most sperm were considered morphologically normal (x̅ = 63.3 ± 23.4%) with the exception of two samples in which morphologically normal sperm accounted for less than 50% of the sperm population (MNW18044, x̅ = 12.5%; MSW18249, x̅ = 48%). Detached heads (x̅ = 16 ± 14%) and coiled/bent midpieces (x̅ = 12 ± 18.8%) accounted for the most common morphological abnormalities across all samples followed by head abnormalities (x̅ = 4.9 ± 4.1%), distal cytoplasmic droplets (x̅ = 3.9 ± 8.6%), coiled/bent tails (x̅ = 2.3 ± 1.2%), and proximal cytoplasmic droplets (x̅ = 0.06 ± 0.2%). Some of the morphological abnormalities observed during morphological analysis with SpermBlue are illustrated in Figure 7.

**TABLE 4:** Mean morphology counts for each individual sample

Animal ID	Morphology						
	Normal	Abnormal head	Detached head	Proximal cytoplasmic droplet	Distal cytoplasmic droplet	Coiled/bent midpiece	Coiled/bent tail
MNW18044	12.5	11	26.5	0	0	59.5	1
MSE17035	82	0.5	6	0	0	8.5	3
MSW17121	59	5.5	3.5	0	26.5	4	3
MSW18221	82.5	0.5	8	0	4	2	3.5
MSW18245*	91.5	2	2.5	0	1	1.5	2.5
MSW18248	63	6	19	0.5	2.5	8	3.5
MNW18103*	67	11	20.5	0	0.5	2	0.5
MSW18249	48	1.5	46	0	1	2.5	2.5
SWFTm1831b*	64	6.5	12	0	0	20	1
Mean	63.3 ± 23.4	4.9 ± 4.1	16 ± 14	0.06 ± 0.2	3.9 ± 8.6	12 ± 18.8	2.3 ± 1.2
Range	12.5–91.5	0.5–11	2.5–46	0–0.5	0–26.5	1.5–59.5	0.5–3.5

*Note*: Mean values are reported as mean ± *SD*. All values are presented as percentages. * indicates samples in which one or more sperm with two tails were observed. This abnormality was not counted in the “coiled/bent tail” category, but should be acknowledged as an additional morphological abnormality observed in Florida manatee sperm.

**FIGURE 8:**
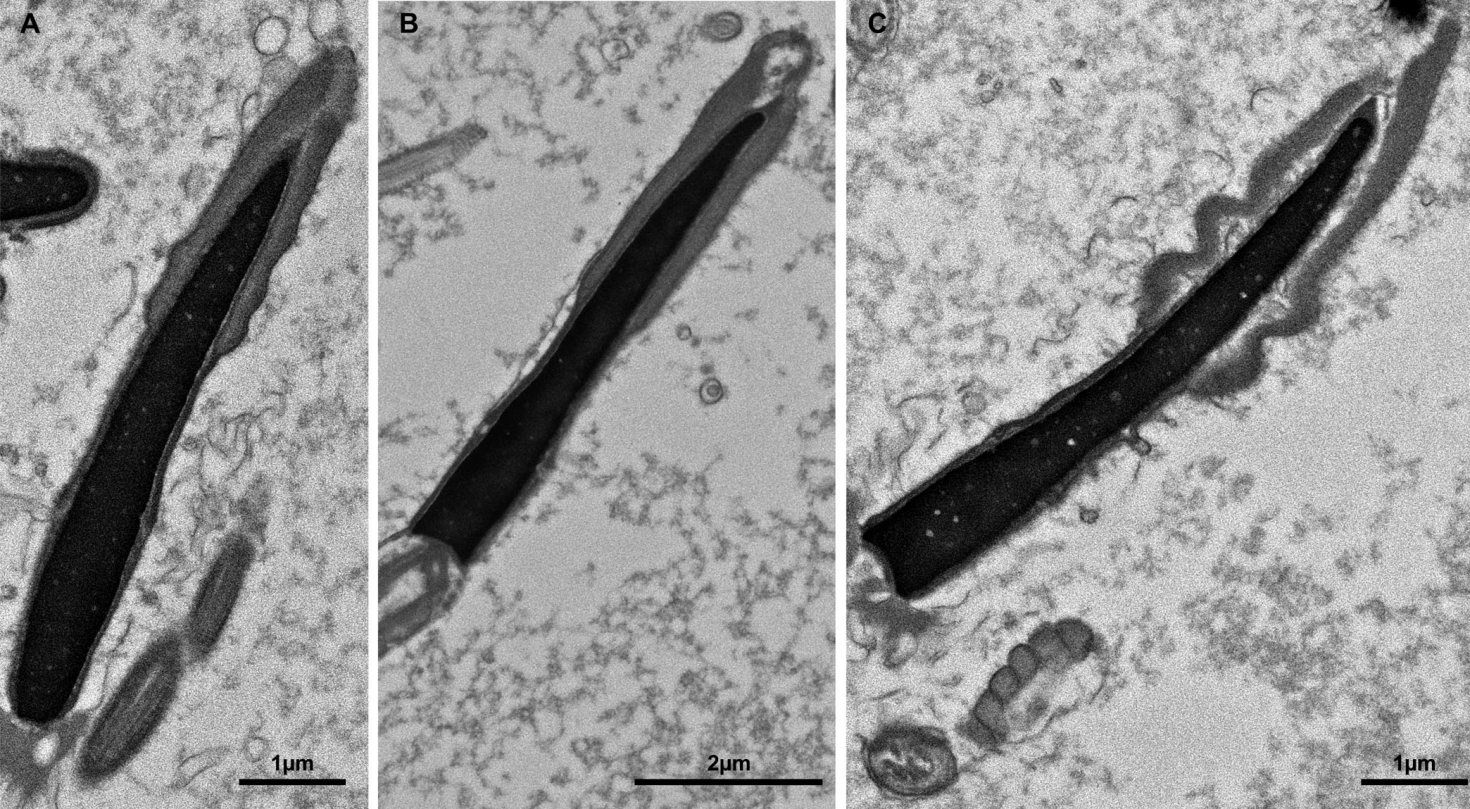
Transmission electron micrographs of Florida manatee sperm heads exhibiting acrosomal defects. (a) Spermatozoon head with degraded plasma membrane and acrosome with minor ruffling. (b) Spermatozoon head with mostly intact plasma membrane and slight dissolution of the apical acrosomal segment. (c) Spermatozoon head with intense acrosomal ruffling, separation of the acrosome from the nucleus in the apical and main acrosomal segments, and dissolution of the external plasma membrane. Acrosomal defects may be the result of postmortem degradation

**FIGURE 9:**
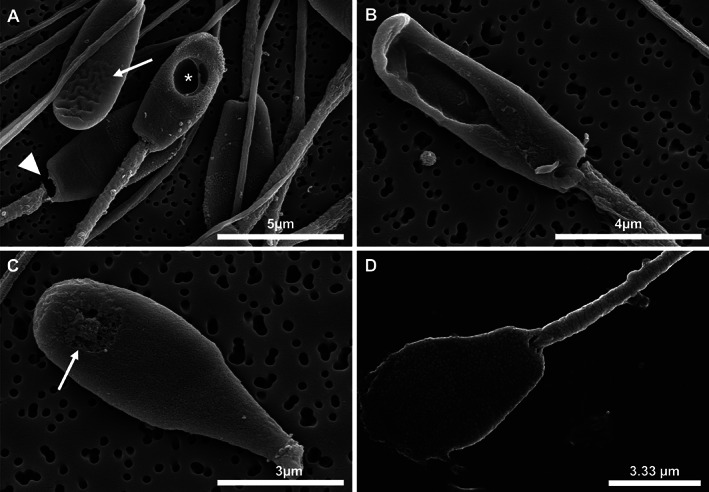
Scanning electron micrographs of Florida manatee sperm head defects. (a) Acrosomal ruffling (arrow), large intranuclear vacuole (crater; asterisk), and separation of spermatozoon head from midpiece (block arrow). (b) Elongation of the spermatozoon head with large intranuclear vacuole. (c) Tapering of the distal end of the spermatozoon head (tapered head shape) with formation of intranuclear vacuole in the apical region (arrow). (d) Abnormal pyriform head shape

**FIGURE 10:**
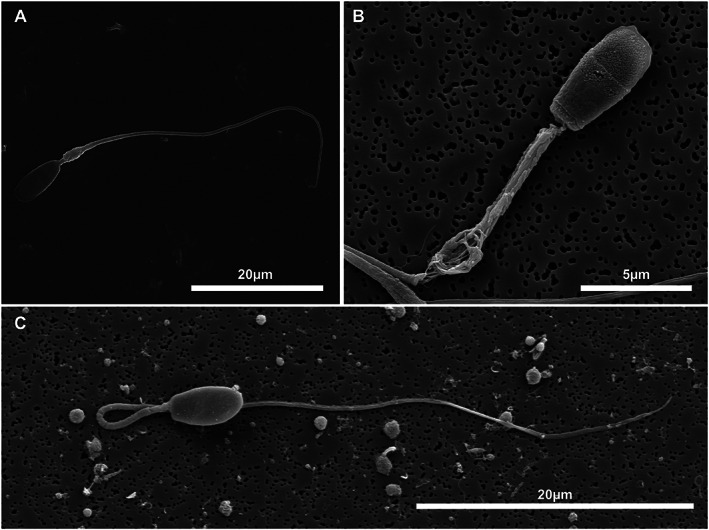
Scanning electron micrographs of Florida manatee sperm midpiece defects. (a) Proximal cytoplasmic droplet (immature sperm). (b) Roughening of the midpiece and degradation of posterior portion of the midpiece at the site of a distal cytoplasmic droplet. (c) Bent midpiece

**FIGURE 11:**
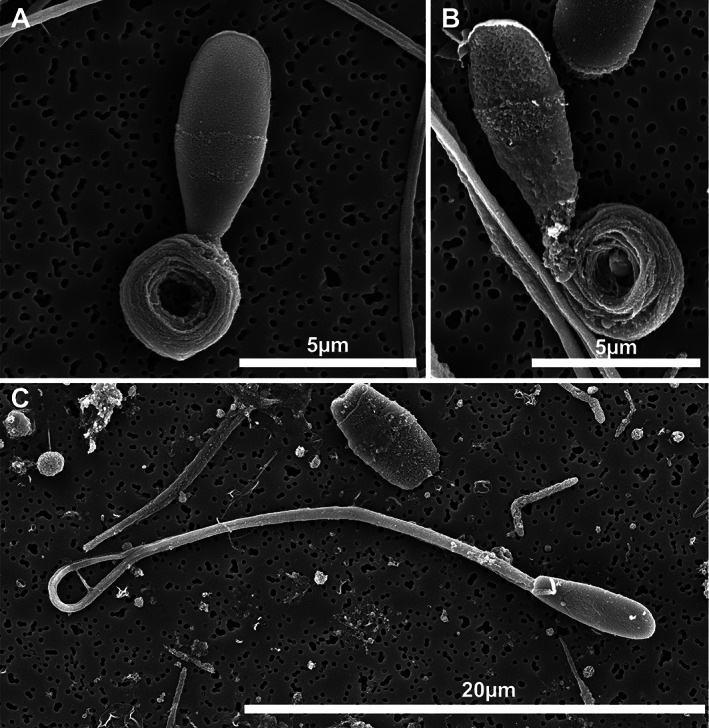
Scanning electron micrographs of Florida manatee sperm flagellar abnormalities. (a, b) Dag defect of midpiece. (c) Hairpin loop at the distal end of the flagellum

At the electron microscope level, abnormalities affecting each component of the spermatozoon were observed. In some samples, acrosomal defects, such as ruffling and dissolution of the acrosome, were observed (Figure 8). Observed head abnormalities included large nuclear vacuoles (nuclear craters), separation of the head and midpiece, tapering of the distal end of the head, and deformation of the head (Figure 9). Observed midpiece abnormalities included proximal cytoplasmic droplets, roughened midpieces from accumulation of cytoplasmic‐like material, degradation of the midpiece, and bent midpieces (Figure 10). Observed flagellar abnormalities included coiling, bending, and hairpin loops (Figure 11).

## 4 DISCUSSION

This study provides the first extensive analysis of sperm morphometry and ultrastructure in the Florida manatee. Updated morphometric values were reported for each component of the Florida manatee spermatozoon. Additionally, many ultrastructural and cytoskeletal elements of the Florida manatee spermatozoon were reported for the first time, thus providing new, functional insight on the structural organization of the Florida manatee spermatozoon.

The morphometric values of this study were approximately 1.5–2 times larger than those reported in Miller et al. (2001) for Florida manatee sperm. Considering there was congruence in the morphometrics obtained using CASMA and SEM, we are confident that the automated software used in this study yielded accurate results. In comparison to other sirenian species, the morphometric values reported herein were most similar to those reported for the Amazonian manatee (*Trichechus inunguis*; Amaral, Lucci, Rosas, da Silva, & Báo, 2010), but differed slightly from those reported for the dugong (*Dugong dugon*; Marsh, Heinsohn, & Glover, 1984).

The ultrastructural results of this study shared many similarities with features previously reported in sirenians (Amaral et al., 2010; Miller et al., 2001); however, some differences were observed from previous descriptions and additional structural components were described for the first time for this species. The head was ovate in shape and dorsoventrally flattened with an extensive acrosome that covered approximately 75% of the spermatozoon head. The midpiece exhibited a helical pattern of mitochondrial gyres terminating at the annulus at the distal end of the midpiece. However, in contrast to that described by Miller et al. (2001), no pronounced annulus was observed in any of the analyzed samples. Our results are in accordance with those reported for the Amazonian manatee (Amaral et al., 2010) in which the annulus is not a prominent or pronounced feature of sirenian sperm.

A unique feature observed consistently across all samples was the distinct enlargement of outer dense fibers (ODFs) 1, 5, 6, and 9. While differences in the shape and size of ODFs are not uncommon across mammalian taxa, enlargement of ODFs is generally limited to ODFs 1, 5, and 6 with a much smaller number of species exhibiting additional enlargement of ODF 9 (Fawcett, 1970; Gu, Zhao, Wang, & Sun, 2019; Olson & Sammons, 1980). Moreover, the morphological form of the enlarged fibers is rectangular‐shaped and seems to be a unique feature for the Florida manatee or potentially sirenians in general. The ODFs are accessory structures to the axoneme that function to protect the flagellum against shear forces as well as provide stabilization to the axoneme and increased stiffness of the flagellum (Gu et al., 2019; Zhao et al., 2018). Thus, relative ODF size plays a major role in velocity and flagellar beating pattern through the transmission of stress along the flagellum and its influence on the bending torque of the flagellum (Lindemann, 1996; Zhao et al., 2018). The enlargement of ODF 9 is a previously unreported feature of Florida manatee sperm and it is currently unclear if this is shared across all sirenians. While not explicitly discussed, Figures 7 and 8 from Amaral et al. (2010) show enlargement of ODFs in the Amazonian manatee. Figure 7 shows enlargement of ODFS 1, 5, and 6 while Figure 8 shows potential enlargement of ODFs 1, 5, 6, and 9 leading us to speculate that this is most likely a shared feature of sirenian sperm, although further investigation is needed to confirm this. The specific function it serves in the Florida manatee (and other sirenians) remains unknown, but it is possible that sexual selective pressures, such as sperm competition, have influenced the structure of the Florida manatee spermatozoon through increased enlargement of ODFs. This may function to further stabilize the flagellum and enhance sperm velocity, thus providing the competitive advantage of increased motility and ability to withstand shear forces associated with navigating the female reproductive tract. Therefore, we hypothesize that the enlargement of four ODFs (rather than three) in the Florida manatee spermatozoon may be related to the potential occurrence of sperm competition in this species.

In addition to enlargement of four ODFs, attention should be paid to the size and structure of the midpiece and flagellum of the Florida manatee spermatozoon. Both midpiece volume and flagellum length have remained controversial sperm traits as it has been argued that a positive relationship should exist between sperm size and velocity (Firman & Simmons, 2010; Immler & Birkhead, 2006). Each trait has a predicted role in this relationship: longer flagellum length results in greater sperm velocity (Katz & Drobnis, 1990) and larger midpiece volume (whether from increased mitochondrial numbers or larger sized mitochondria) results in greater power output (Cardullo & Baltz, 1991). Unfortunately, comparative studies have produced conflicting results and there appears to be a species‐specific nature to this relationship. For the Florida manatee, it is unclear if the sexual selective pressures of a promiscuous mating system and the predicted occurrence of sperm competition have influenced the size or structure of the midpiece and/or flagellum. In many species, a correlation between midpiece volume (rather than midpiece length) and mating system has been documented. Genera that utilize multi‐partner mating systems have significantly larger midpiece volumes in comparison to genera with single partner mating systems (Anderson & Dixson, 2002; Anderson, Nyholt, & Dixson, 2005). Preliminary measurements of midpiece volume (as per Anderson et al., 2005) for the Florida manatee yielded an average of 4.23 μm^3^. This midpiece volume aligns more closely with species that exhibit a multi‐partner mating system and provides further evidence for the possible occurrence of sperm competition within this species. Continued research is necessary to find meaningful correlations between both morphometric (midpiece volume and flagellum length) and structural components (enlargement of four ODFs) and the mating system of the Florida manatee.

The structural proteins F‐actin and alpha‐tubulin were localized within the Florida manatee spermatozoon. F‐actin filaments had the strongest localization within the midpiece that decreased throughout the flagellum. Similar high intensity localization patterns of F‐actin within the spermatozoon midpiece has been observed in other species ([rat, mouse, guinea pig] Flaherty, Winfrey, & Olson, 1986; [mouse] Bouchard et al., 2000). Recent studies in mice show a double‐helical structure of F‐actin in conjunction with the helical structure of the mitochondria, which suggests F‐actin may act as a structural component for supporting the integrity of the mitochondrial sheath and thus promoting normal sperm function and maintaining sperm integrity (Gervasi et al., 2018). The similar staining pattern of F‐actin observed in the Florida manatee spermatozoon may suggest a similar structural configuration as that of mice. Therefore, F‐actin may be an important structural element of the Florida manatee spermatozoon and may play a major functional role in maintaining sperm integrity within the female reproductive tract. Alpha‐tubulin localization was also strongest within the sperm midpiece and throughout the flagellum, highlighting the identification of the microtubules of the sperm axoneme. Alpha‐tubulin was also faintly localized along the equatorial region of the sperm head. The function of alpha‐tubulin in the Florida manatee sperm head is unknown but it may represent an important cytoskeletal component for sperm head morphology in terms of providing support and potentially signal transduction mechanisms.

The morphological abnormalities observed across all samples in this study were similar to those observed in other mammalian species. In most samples, sperm with normal morphology exceeded 60% of the analyzed sperm population with two of the nine samples exceeding 80%. This potentially indicates that most males analyzed in this study had good fertility potential given the large percentage of morphologically normal sperm observed across the samples. However, it is important to consider the effects of postmortem degradation of sperm in the Florida manatee, which may represent a confounding variable in samples with higher levels of morphologically abnormal sperm. Investigation into the effects of postmortem degradation on sperm is necessary to determine optimal conditions for postmortem sperm retrieval and analysis in the Florida manatee. Regardless, the morphological information gained in this study provides a solid foundation for continued reproductive research in this species.

Overall, this study represents the first application of CASMA in any sirenian species and highlights the effectiveness of this technology for studying sperm morphometry in endangered and exotic species. The morphometric and ultrastructural results provide new functional insights on the structural organization of the Florida manatee spermatozoon. These features not only provide baseline information for future reproductive research and conservation of this species, but also offer preliminary insight into the potential effects of sexual selective pressures on sperm morphology and structure as well as evidence for the possible occurrence of sperm competition in the Florida manatee. Future research is necessary to determine how sexual selective pressures have influenced sperm size and structure in this species and their relation to the mating system exhibited in Florida manatees and across sirenian species.

## AUTHOR CONTRIBUTIONS

**Jonathan Cowart:** Conceptualization; data curation; formal analysis; funding acquisition; investigation; project administration; resources; visualization; writing‐original draft. **Danielle Collins:** Conceptualization; formal analysis; investigation; writing‐review & editing. **Daniel Stanton:** Formal analysis; investigation; writing‐review & editing. **Gerhard van der Horst:** Conceptualization; writing‐review & editing. **Iskande Larkin:** Funding acquisition; project administration; resources; writing‐review & editing.

## CONFLICT OF INTEREST

Gerhard van der Horst is a senior consultant for Microptic SL (Barcelona, Spain), which manufactures the SCA computer‐aided sperm analysis system software used in this study. However, all analyses were performed independently and Microptic SL had no bearing on the outcome.

## ETHICS STATEMENT

This study was reviewed and approved by the University of Florida Institutional Animal Care and Use Committee (UF IACUC protocol #20180884). All samples were received and analyzed under Federal Fish and Wildlife Permit #MA067116‐2.

## Data Availability

The morphometric datasets generated and analyzed during the current study are openly available in the institutional repository of the University of Florida (IR@UF) and can be accessed at: https://ufdc.ufl.edu//IR00011368/00001. Necropsy data are publicly available and can be requested directly from the Florida Fish and Wildlife Conservation Commission Marine Mammal Pathobiology Laboratory.
